# A Rare Case of COVID-19 Associated With Autoimmune Hemolytic Anemia, Thrombocytopenia and Acute Kidney Injury

**DOI:** 10.7759/cureus.26010

**Published:** 2022-06-16

**Authors:** Muzamil Musa, El Mustafa Abdalla, Maab F Elhaj, Salma Mustafa, Sharif A Ahmed, Jamal Sajid

**Affiliations:** 1 Internal Medicine, Hamad Medical Corporation, Doha, QAT; 2 General Practice, Communicable Disease Center - Hamad Medical Corporation, Doha, QAT; 3 Medicine, Hamad General Hospital, Doha, QAT

**Keywords:** hemolytic anemia, covid 19, acute kidney injury, aiha, thrombocytopenia, infection

## Abstract

Severe acute respiratory syndrome coronavirus 2 (SARS-CoV-2) has numerous effects on different systemic organs other than the lungs. In this case report, we look at the presentation of a young female who was diagnosed with autoimmune hemolytic anemia (AIHA), kidney injury and thrombocytopenia during coronavirus disease 2019 (COVID-19) infection. She recovered well without the need for steroids. As demonstrated by this case, COVID-19 infection can be associated with the development of AIHA. The purpose of this report is to indicate that COVID-19 can present unusually with different clinical manifestations enough to require hospitalization.

## Introduction

Information from the medical literature on coronavirus disease 2019 (COVID-19) infection and its hematological consequences is rapidly growing. COVID-19 is mostly associated with respiratory illness and complications. However, the virus can result in multi-organ failure as a result of its pathology [1]. To our knowledge, this is one of the few cases of relatively healthy patients presenting with COVID-19 and autoimmune hemolytic anemia (AIHA). Here, we present the case of a lady who deteriorated dramatically during her admission as a result of COVID-19 infection. This case adds to the evidence for the association between AIHA and COVID-19 and will help in raising awareness of the unusual symptomatology of COVID-19.

## Case presentation

A 32-year-old Middle Eastern female with a history of multiple sclerosis presented with fever for two days to the emergency department. There were no other associated symptoms. She was on teriflunomide and tizanidine for multiple sclerosis. She did not receive any COVID-19 vaccination. Upon initial assessment, her vital signs were within normal range with oxygen saturation of 98% on room air, and systemic examination was unremarkable. The polymerase chain reaction (PCR) swab for COVID-19 was positive. Here chest x-ray showed no abnormalities in lung fields. Laboratory findings were significant for leukocytosis with a white blood cell (WBC) count of 14.5 x 10^3^/uL, while others including inflammatory markers and complete metabolic profile were unremarkable. Since she was not hypoxic, according to our local protocol, she did not qualify to receive any steroids or antiviral medications.

**Figure 1:**
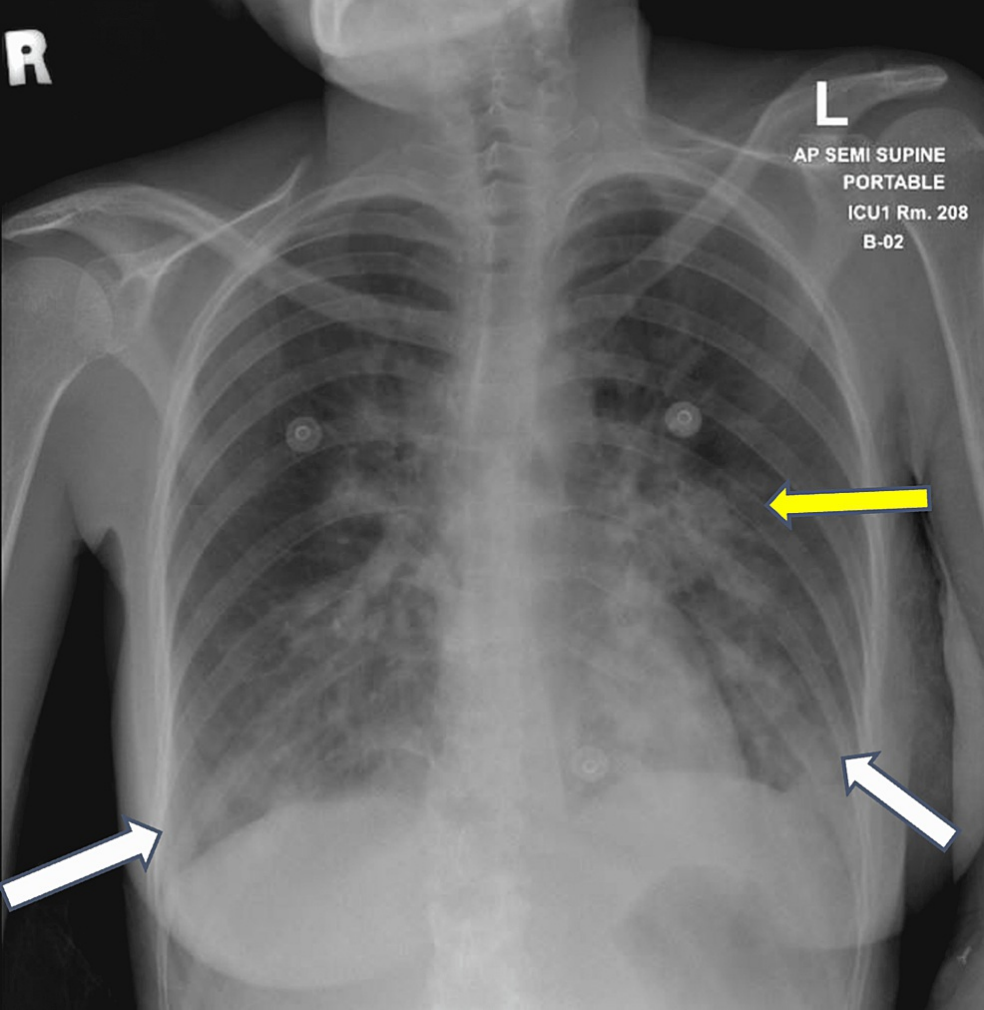
Chest x-ray showing findings of pneumonic consolidation in the bilateral lower lung zone (white arrows) and left-sided perihilar region (yellow arrow)

On the third day of hospital admission, she became dyspneic, tachycardic and hypoxic. The electrocardiogram (ECG) showed normal sinus rhythm and troponins were negative. Chest x-ray was repeated and showed bilateral lower lung zone opacities (Figure 1). She was transferred to the intensive care unit (ICU) as a case of moderate-severe COVID-19 infection and was managed with nasal cannula only and did not require invasive ventilation.

During her ICU stay, her hemoglobin and platelet levels dropped to 8.0 gm/dL and 105 x 10^3^/uL, respectively (both within normal range initially) and subsequent blood tests were suggestive of hemolysis with a lactate dehydrogenase (LDH) level of of 583 U/L (reference, 135-214 U/L), haptoglobin level of <10 mg/dL (reference, 30-200 mg/dL) and reticulocyte count of 4.2% (reference, 0.5%-2.5%). A blood smear showed polychromasia with spherocytes suggestive of hemolytic anemia. The direct antiglobulin test (DAT) revealed significant levels of immunoglobulin G (IgG) anti-RBC autoantibodies. The Coombs profile confirmed warm-type autoimmune hemolytic anemia. In her other blood tests, ferritin was increased from 458 to 13,090 ng/mL (reference, 12-300 ng/mL). Additionally, she had an acute kidney injury (AKI) with creatinine 157 umol/L (reference, 44-80 umol/L). Ultrasound showed no renal stones or hydronephrosis, and spleen and liver were normal. A tentative diagnosis of thrombotic thrombocytopenic purpura (TTP) was ruled out on the basis of a low reticulocyte count and the positive direct Coombs test.

The patient was negative for infectious diseases (human immunodeficiency virus, hepatitis A/B/C, cytomegalovirus, Epstein-Barr virus, syphilis, and *Mycoplasma pneumoniae*). Her autoimmune workup was negative, and her vitamin B12 and glucose-6-phosphate dehydrogenase levels were normal. A summary of the investigation results during hospital stay is shown in Table 1.

**Table 1:** Blood investigations during hospitalization WBC, white blood cell; BUN, blood urea nitrogen; CRP, C-reactive protein; LDH, lactate dehydrogenase; AST, aspartate aminotransferase; ALT, alanine aminotransferase

Reference range	Admission	Day 3	Day 7	Discharge
Hemoglobin (13-15 g/dL)	12	8	9.4	10
WBC (4-10 K/uL)	14	15		5
Platelets (150-450 K/uL)	220	105	76	140
BUN (2.5-7.8 mmol/L)	3	8.2	9	6.5
Creatinine (44-80 umol/L)	62	157	340	76
CRP (0-5 mg/L)	11	25	6	2
Haptoglobin (30-200 mg/dL)		<10	<10	
Procalcitonin (<0.5 ng/mL)	0.3	0.5	0.5	0.1
Ferritin (12-160 ug/L)	458	13,090	238	140
LDH (145-214 u/L)	170	583	853	130
Bilirubin (total) (0-21 umol/L)		54	60	32
AST (0-32 u/L)	44	42	40	30
ALT (0-33 u/L)	30	33	32	35
Reticulocyte count (0.5%-2.5%)		4.2%		2.9%

For next few days, her hemoglobin level and platelets continued to drop, and creatinine level continued to rise. She was given blood transfusions for anemia and required multiple sessions of hemodialysis for renal failure. We held a multidisciplinary meeting with the nephrologist and hematologist, and it was decided not to give her any steroids at that stage. After another few days, her hemoglobin, platelets, LDH, bilirubin, reticulocyte count and creatinine started to improve without any specific treatment. She was safely discharged home on folic acid after few more days of observations.

## Discussion

AIHA is a unique immune condition caused by anti-RBC autoantibodies destroying red blood cells with or without the activation of the complement system [2]. With respect to its etiology, it can occur in association with several autoimmune disorders, neoplasia, infectious diseases or medications [3].

According to the literature, AIHA and COVID-19 infection have previously been linked to either warm or cold autoantibodies [4]. Lazarian et al. found that nine days was the median period between the development of AIHA and the first COVID-19 clinical features [5]. In our case report, AIHA occurred three days after the beginning of COVID-19 symptoms. According to Quinn and Murakhovskaya, a great proportion of the cases were the warm type of AIHA with the remainder being cold [6]. Similarly, our case also exhibited a positive IgG antiglobulin test. In another report, Hindilerden et al. described a case in which there was an association between the two diseases [7]. In contrast, in other cases, the patients suffered from malignant or lymphoproliferative disorders [5]. Our patient did not have any underlying neoplastic or hematological concomitant disorders.

The underlying pathophysiologic molecular mechanism in virus-induced cell death is not well known; however, it is thought to be an autoimmune reaction through antigenic mimicry, in which autoantibodies destroy self-antigens [8]. After ruling out all other possibilities, we can proclaim virus-induced AIHA as the culprit.

Acute renal impairment has been linked to an elevated death rate in COVID-19 hospitalized patients, with an 8.9% incidence reported in a recent meta-analysis [9,10]. The mechanism of this issue is not well known. Studies have shown that the pathogenesis is multifactorial, and both direct and indirect mechanisms are responsible for COVID-19-related AKI [11].

Thrombocytopenia was recently recognized as a common feature of the COVID-19 pandemic and was found in up to 36% of people [9]. It is also associated with high mortality and severe disease. One of the published studies in May 2020 reported a patient with COVID-19 who had no pulmonary abnormalities but significant thrombocytopenia. It illustrates the virus's abnormal presentation, which might range from asymptomatic cases to those with unexpected laboratory results [12].

From the management point of view, in the case of inability to treat the underlying cause, steroids remain the first-line treatment of choice for secondary AIHA patients [13]. Rituximab has been reported to be given following inadequate response to corticosteroids in only one case [5]. We did not use steroids for our patient as she was recovering without the need for them and her deterioration was reversible, and she had stable hemoglobin levels on her subsequent follow-ups.

## Conclusions

There have been few reports of patients with concurrent COVID-19 and AIHA and the association remains to be elucidated. This serves as a reminder for clinicians that when encountering unexplained anemia in patients with a history of COVID-19, AIHA should be considered. It also highlights that treating the cause of secondary AIHA can improve patient condition without the need for steroids. More studies need to be done to establish the relationship between COVID-19 and AIHA.
